# Proline and ROS: A Unified Mechanism in Plant Development and Stress Response?

**DOI:** 10.3390/plants14010002

**Published:** 2024-12-24

**Authors:** Marco Renzetti, Dietmar Funck, Maurizio Trovato

**Affiliations:** 1 Department of Biology and Biotechnology, Sapienza University of Rome, 00185 Rome, Italy; renzetti.1818304@studenti.uniroma1.it; 2 Department of Chemistry, University of Konstanz, 78464 Konstanz, Germany; dietmar.funck@uni-konstanz.de

**Keywords:** proline metabolism, root meristem size, ROS, Arabidopsis, *prodh1 prodh2*, *p5cs1 P5CS2/p5cs2*, hydrogen peroxide, antioxidant enzymes

## Abstract

The proteinogenic amino acid proline plays crucial roles in both plant development and stress responses, far exceeding its role in protein synthesis. However, the molecular mechanisms and the relative importance of these additional functions of proline remain under study. It is well documented that both stress responses and developmental processes are associated with proline accumulation. Under stress conditions, proline is believed to confer stress tolerance, while under physiological conditions, it assists in developmental processes, particularly during the reproductive phase. Due to proline’s properties as a compatible osmolyte and potential reactive oxygen species (ROS) scavenger, most of its beneficial effects have historically been attributed to the physicochemical consequences of its accumulation in plants. However, emerging evidence points to proline metabolism as the primary driver of these beneficial effects. Recent reports have shown that proline metabolism, in addition to supporting reproductive development, can modulate root meristem size by controlling ROS accumulation and distribution in the root meristem. The dynamic interplay between proline and ROS highlights a sophisticated regulatory network essential for plant resilience and survival. This fine-tuning mechanism, enabled by the pro-oxidant and antioxidant properties of compartmentalized proline metabolism, can modulate redox balance and ROS homeostasis, potentially explaining many of the multiple roles attributed to proline. This review uniquely integrates recent findings on the dual role of proline in both ROS scavenging and signaling, provides an updated overview of the most recent research published to date, and proposes a unified mechanism that could account for many of the multiple roles assigned to proline in plant development and stress defense. By focusing on the interplay between proline and ROS, we aim to provide a comprehensive understanding of this proposed mechanism and highlight the potential applications in improving crop resilience to environmental stress. Additionally, we address current gaps in understanding and suggest future research directions to further elucidate the complex roles of proline in plant biology.

## 1. Introduction

Plants are sessile organisms and therefore have to coordinate the completion of their life cycle with whatever environmental conditions they face. Both developmental decisions and adaptive or defense responses are therefore regulated by a multitude of environmental and internal cues. Accumulation of free proline in excess of the need for protein synthesis has traditionally been associated with stress tolerance but is now emerging additionally as a crucial factor in plant development. The multifunctional nature of proline makes this amino acid a key mediator in stress-related responses, including osmoprotection, protection of proteins and membranes, antioxidant defense, protein folding, and signal transduction [1]. In addition, proline has been involved in several developmental processes such as floral transition [2,3,4,5], pollen fertility [6,7], embryo development [8,9], cell wall synthesis [10], and root elongation [11,12,13].

Proline metabolism or its accumulation is believed to counteract dehydration, stabilize proteins and membranes, scavenge free radicals, maintain cellular homeostasis, buffer redox environment, trigger signal transduction, and affect gene expression. The underlying molecular mechanisms and the relative importance of these multiple functions, however, are still not fully understood. In order to use modification of proline metabolism for crop fortification, further investigations of its contributions to the regulatory network of adaptive and developmental processes are needed. Although several excellent papers have separately addressed the importance of ROS in plants [14,15,16,17,18] and their interactions with proline under stress [19,20] and non-stress conditions [13], the possibility that a single, or predominant, mechanism may control the responses to proline under stress and non-stress conditions has never been discussed. This review aims to fill this gap by addressing the importance of proline–ROS interactions, exploring their role in plant development and stress response, and discussing the possibility that proline control of redox balance and ROS signaling may underlie most of the multiple functions attributed to proline.

## 2. The State of the Art: Multiple Roles of Proline in Stress and Development

### Proline in Stress Responses

**Figure 1:**
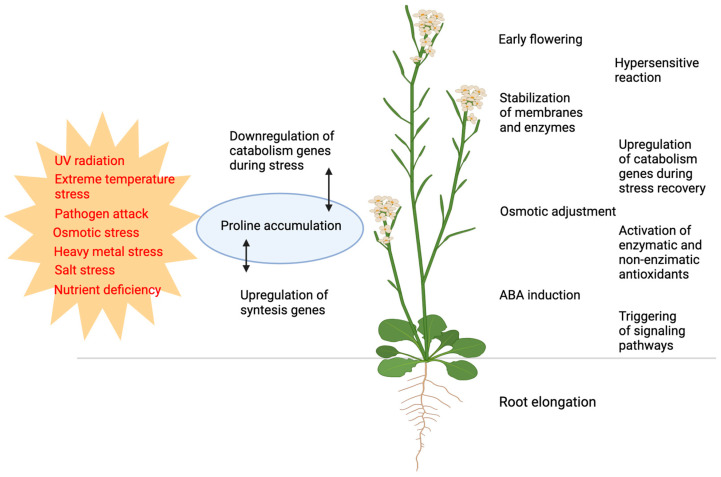
Functions of proline and proline metabolism in responses to biotic and abiotic stresses. Various types of biotic and abiotic stresses (shown in red) induce changes in proline metabolism, most frequently resulting in proline accumulation (blue). The alterations in proline metabolism result in or are correlated to a broad range of defense responses (shown in black). Figure created with BioRender.com.

Historically, the primary role of free proline accumulation has been associated, ever since the middle of the last century, with its protective effects against a number of biotic and abiotic stresses, including salinity, drought stress, extreme temperature, water stress, heavy metal stress, UV light stress, nutrient deficiency, and pathogen attacks [1,21,22,23,24,25] (Figure 1). However, compelling evidence for the beneficial effects of proline accumulation was only provided in 1981, specifically in bacterial tolerance to osmotic stress [26].

The main reasons for the popularity of proline as a potential stress protector were the peculiar chemical properties of proline and its rapid and abundant accumulation observed under stress conditions in many plant species [22,23,25,27]. Indeed, although some plant species can respond to stress by accumulating other types of osmolytes, such as glycine betaine, γ-aminobutyric acid (GABA), trehalose, mannitol, sorbitol, and polyamines, among others [28], proline accumulation is the most widespread among plant species, as well as in various organisms such as protozoa, eubacteria, marine invertebrates, and algae [29,30,31,32].

**Figure 2:**
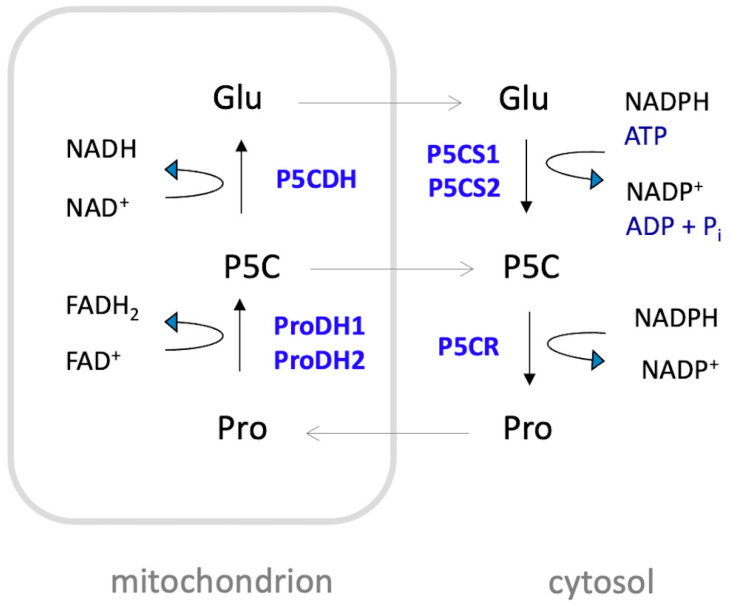
Schematic representation of proline metabolism in *Arabidopsis*. Proline (Pro) is mainly synthesized in the cytosol from glutamate (Glu) by a two-step reduction catalyzed by Δ1-pyrroline-5-carboxylate synthetase (P5CS) and Δ1-pyrroline-5-carboxylate reductase (P5CR). The process involves the consumption of one molecule of ATP and the oxidation of two molecules of NADPH to NADP+. Proline catabolism takes place in the mitochondrion, where proline is oxidized to Δ^1^-pyrroline-5-carboxylate (P5C) by the action of ProDH1 and ProDH2, with concomitant reduction of FAD to FADH_2_. Subsequently, P5C is oxidized to glutamate via P5C dehydrogenase (P5CDH), coupled with the reduction of NAD^+^ to NADH. In *Arabidopsis*, P5CS and ProDH are encoded by two pairs of paralogous genes: *P5CS1* and *P5CS2*, and *ProDH1* and *ProDH2*, respectively. These genes encode the isozymes P5CS1, P5CS2, ProDH1, and ProDH2.

The extent of proline accumulation under adverse conditions depends on the plant species and can increase, in strong accumulators, by several hundred-fold compared to unstressed levels [33]. Proline is primarily synthesized in the cytosol from glutamate through a two-step reduction process (Figure 2). This process is catalyzed by Δ^1^-pyrroline-5-carboxylate synthetase (P5CS) and Δ^1^-pyrroline-5-carboxylate reductase (P5CR). During the first step, P5CS catalyzes the conversion of glutamate to glutamate-γ-semialdehyde (GSA), which is in tautomeric equilibrium with Δ^1^-pyrroline-5-carboxylate (P5C). This step involves the consumption of ATP and is coupled with the oxidation of NADPH to NADP^+^. In the second step, P5CR catalyzes the reduction of P5C to proline, also coupled with the oxidation of NAD(P)H to NAD(P)^+^. An alternative route from ornithine is also possible in animals and fungi, but it seems to be prevented in plants due to the localization of ornithine aminotransferase and P5CR in separate cell compartments [34,35,36]. Proline catabolism occurs in the mitochondria, where ProDH catalyzes the oxidation of proline to P5C, reducing FAD to FADH_2_, and P5CDH catalyzes the conversion of P5C to glutamate, reducing NAD^+^ to NADH (for a thorough review on proline metabolism see Trovato et al., 2019 [37]).

The electrons from FADH_2_ and NADH are transferred via the electron transport chain to molecular oxygen, generating a proton gradient across the inner mitochondrial membrane, which drives ATP synthesis via ATP synthase. However, as a by-product of mitochondrial respiration, superoxide anions (O_2_^•–^) are continuously generated and can cause oxidative damage to cellular components unless they are quenched by the antioxidant defense system [38]. In the mitochondrion, O_2_^•–^ is rapidly converted to hydrogen peroxide (H_2_O_2_) by the action of superoxide dismutases (SOD). H_2_O_2_ can then pass through the mitochondrial membrane and diffuse into the cytosol or even neighboring cells, where it can act as either a signaling molecule activating the cellular antioxidant defense and further adaptive responses or a toxic agent leading to oxidative damage and death [38]. Depending on the amount of H_2_O_2_ produced, proline catabolism can therefore induce antioxidant or pro-oxidant responses.

At the molecular level, stress triggers the rapid transcriptional upregulation of *P5CS*, the gene encoding the rate-limiting enzyme in proline biosynthesis, and possibly *P5CR*, which catalyzes the second committed step in this pathway. In *Arabidopsis*, P5CS is encoded by the paralogous genes *P5CS1* and *P5CS2*, and only *P5CS1* is rapidly induced by stress through ABA-dependent and ABA-independent mechanisms, while *P5CS2* has housekeeping and developmental functions [8,9,39,40,41]. Duplication of *P5CS* genes has occurred independently in several plant lineages and the functional specialization appears similar, although not identical, in different plant species [42,43,44]. While proline accumulation in the cytosol is largely driven by enhanced biosynthesis, it is further promoted by the downregulation of proline dehydrogenase (*ProDH*), the gene coding for the rate-limiting enzyme in proline catabolism, and possibly Δ^1^-pyrroline-5-carboxylate dehydrogenase (*P5CDH*), which codes for the second enzyme of proline degradation. In *Arabidopsis*, ProDH is encoded by two paralogous genes, *ProDH1* and *ProDH2*. The downregulation of the expression of these genes reduces proline degradation during stress [33].

Chemically, proline has unique properties that make it important in protein folding and effective as a compatible osmolyte. Structurally, it is the only amino acid in which the α-amino group is reconnected to the side chain to form a secondary amine in a heterocyclic structure. This conformation confers exceptional structural rigidity to the protein structure and makes it important in protein folding [45,46]. It is often found in polyproline- or hydroxyproline stretches in cell wall proteins, such as extensins, and arabinogalactan proteins, which are thought to play key roles in cell elongation and signal transduction [47]. In addition, free proline is a zwitterionic molecule with a pK_a_ in the neutral pH range, high compatibility with cellular metabolism, and high solubility in water, where it can reach concentrations of up to 6.5 M [48], making this amino acid a perfect candidate as a compatible osmolyte.

While clear evidence of proline conferring stress tolerance in plants is still debated, numerous transgenic plants have been engineered over the years to alter proline metabolism, by overexpressing proline biosynthesis genes, downregulating proline catabolism genes, or increasing transport from one tissue to another. The studies have shown increased stress tolerance, at least under laboratory conditions, supporting the idea that proline accumulation helps plants withstand adverse conditions [49]. Additionally, treatments with proline have been reported to effectively protect crops from various environmental stresses, further supporting its positive role in stress responses [25,50]. However, despite general agreement on proline as a reliable stress marker, consensus on its positive effects under stress conditions is not unanimous. Some researchers have reported neutral or negative effects of proline accumulation under stress [51,52,53,54,55,56], suggesting that in some cases proline might be a mere symptom of stress rather than a cure to relieve it [55].

## 3. Mechanisms of Proline’s Protective Role Under Abiotic Stress

### 3.1. Osmoprotection

Assuming that proline accumulation improves plant tolerance to stress damage, it remains to be understood how and by which molecular mechanisms. Given the peculiar chemical properties of proline as a compatible osmolyte, it is difficult not to believe that these properties are at the basis of proline-induced stress tolerance.

Indeed, the osmoprotective function of proline is one of the earliest and most widely accepted mechanisms to explain the role of proline in stress tolerance [22,57]. Under osmotic stress conditions, such as drought or salinity, the accumulation of osmolytes in the cytoplasm can help reduce the cellular water potential below the external water potential without adversely affecting cellular metabolism. In this way cells limit or stop water loss and preserve turgor pressure. This osmotic adjustment is thought to be critical for protecting cellular structures and maintaining the integrity of membranes and proteins, thus preventing cellular damage and ensuring continued metabolic activity. Since its first formulation, this hypothesis has been confirmed by numerous publications (See Forlani et al., 2019 [33] for a review) and has become broadly accepted as a mechanism to explain proline-induced tolerance to osmotic stress.

However, despite its popularity, this putative mechanism of action has never been conclusively proven and has been questioned by some authors [33,54,58,59,60]. The first doubts were raised in a seminal paper by Hare and Cress (1997) [51], who noted that in most cases the cytoplasmic pool of free proline appeared to be too small to account for a relevant contribution of proline to the overall osmolarity, even during a stressful episode. Later, subsequent studies made on cell suspension cultures have supported this early hypothesis by demonstrating that even under severe osmotic stress, the concentration of proline rarely increases beyond a few micromoles per gram of fresh weight. For instance, some studies that quantified the cellular sap concentration along with its chemical components found that proline’s contribution to the necessary increase in osmolarity was no more than 3 to 15%, even though its concentration had increased by a factor of one hundred compared to the controls [61,62,63]. Along the same line, only modest or no contribution to osmotic adjustment was found in flour wheat (*Triticum aestivum*) [64], barley (*Hordeum vulgare*) [65], and durum wheat (*Triticum turgidum durum*) [66]. Overall, apart from a few exceptions, such as in maize (*Zea mays*) roots grown at low water potentials, where proline accumulation can account for 45% of the total osmotic adjustment [67], or in the halophytes salt cress (*Thellungiella halophila*) and thick-leaved pepperwort (*Lepidium crassifolium*), which accumulate large amounts of proline [68,69], in most glycophytes the level of proline accumulation appears to be insufficient for meaningful osmotic adjustment compared to other osmolytes such as potassium (K^+^) and sodium (Na^+^), which contribute much more to the osmotic pressure of the cell [33]. Moreover, a recent meta-analysis found that the transgenic expression of proline metabolism genes has overall positive effects on drought and salinity tolerance, but only marginally through osmotic regulation [49].

### 3.2. Protection of Proteins and Membranes

Another property of proline thought to be responsible for or contribute to stress tolerance is its capability to protect and stabilize enzymes and membranes from environmental stresses [51,70,71]. It is not clear which mechanism or mechanisms can explain these effects on protein and membrane stability, but obviously, the compatible osmolyte’s properties of proline have the potential to perform this role. With the limitations discussed in the previous paragraph, the effect of proline on overall osmolarity may help cells maintain their volume and water content during periods of environmental stress, preserving proteins and membranes from dehydration. However, considering the controversial contribution of proline in osmotic adjustment, other mechanisms have been proposed to act in substitution to or synergistically with osmotic adjustment, the most convincing of which stems from the kosmotropic properties of proline.

When cells experience osmotic stress, the withdrawal of water can lead to the accumulation of chaotropic substances that disrupt protein structure and function. Proline, similarly to other compatible solutes, exerts a kosmotropic (anti-chaotropic) effect by promoting the ordering of water molecules around macromolecules or by replacing them, which is critical for maintaining proper three-dimensional protein folding and enzymatic activity. This ordered hydration shell reduces entropy, helping to stabilize proteins and prevent unfolding. Furthermore, according to Schobert and Tschesche (1978) [48], proline could increase the hydrophilic surface area of proteins and enhance their water-binding capacity by stepwise stacking, and hydrophobic interactions involving its pyrrolidine ring. This increased hydration capacity has been shown to alleviate the negative effects of dehydration on protein stability and activity [71]. Additionally, proline is believed to facilitate protein renaturation and prevent aggregation by trapping folding intermediates in a supramolecular assembly [72].

In terms of membrane stabilization, proline interacts with phospholipid headgroups, modulating membrane fluidity and permeability [73]. This interaction helps to maintain the structural integrity of cellular membranes during stress, preventing leakage and preserving cellular compartmentalization. Additionally, proline’s ability to form hydrogen bonds with water molecules contributes to the formation of a protective hydration layer around membranes, further enhancing their stability under adverse conditions [74].

### 3.3. ROS Scavenging

Among the functions proposed for proline to explain its beneficial effects on environmental stress, the ROS scavenger has been one of the most accredited but, at the same time, most controversial. In plant cells, ROS are constantly produced as by-products of photosynthetic and mitochondrial electron transport, as well as by peroxisomal metabolism, and the production rates are typically increased by stress [38]. Additionally, NADPH oxidases and peroxidases mediate extracellular production of ROS, which can react in the cell wall or diffuse back into the cells [75,76].

The ROS scavenging function of proline was first reported in the seminal work of Smirnoff and Cumbes [77], who suggested, as early as 1989, that proline could effectively scavenge hydroxyl radicals (•OH) and proposed that proline could react with various ROS, laying the foundation for further research into the antioxidant capabilities of proline. Later, proline was shown to reduce lipid peroxidation as shown by reduced malondialdehyde (MDA) formation, first in brown mustard (*Brassica juncea*) cotyledons under various stress conditions [78,79,80], and subsequently in a number of different species [49,81], confirming the antioxidant effects of proline.

While the protective effects of proline against oxidative damage are generally accepted by the scientific community, the ability of proline to be a direct ROS scavenger is based more on logical considerations—considering that one of the main detrimental effects of stress is the generation of ROS—than on solid evidence, which is mainly based on theoretical and computational studies or in vitro studies. Overall, there is some correlative or theoretical evidence to support the hypothesis that proline can directly scavenge the hydroxyl radical and, perhaps, singlet oxygen (^1^O_2_), but there is no sound evidence for a direct effect on the superoxide anion (O_2_^•–^) and hydrogen peroxide (H_2_O_2_).

For •OH, the conclusions of Smirnoff and Cumbes were confirmed and extended by Signorelli et al. [82], who found by computational chemistry that hydrogen abstraction from proline is the most favored reaction with •OH and yields less reactive intermediates. However, for a true protection mechanism, proline would have to be far more abundant than the compounds or proteins damaged by •OH.

With respect to ^1^O_2_, the original claim that proline is a quencher of singlet oxygen in isolated thylkoids because of its ability to reduce singlet oxygen-mediated 2,2,6,6-tetramethylpiperidin (TEMP) oxidation [83] was refuted by Signorelli et al. [84], who reanalyzed the putative mechanisms of quenching by real-time measurement of singlet oxygen fluorescence and concluded that proline was not able to quench singlet oxygen.

More recently, Rehman et al. [85] employed paramagnetic resonance spin trapping with TEMPD fluorescence probing using singlet oxygen sensor green and oxygen uptake measurements in isolated thylakoids, demonstrating that proline quenches both singlet oxygen and superoxide radicals (O_2_^•−^) in vitro through an electron transfer mechanism. However, these claims are questionable because they have only been demonstrated in in vitro systems and because they are in contrast with the elegant conclusions of Hamilton and Heckathorn [86], who demonstrated that the primary cause of mitochondrial electron transport disruption due to saline stress is oxidative damage in complex I and Na^+^ toxicity in complex II, and that proline was unable to protect complex I. As superoxide dismutase (SOD) provided the highest protection of complex I, the authors concluded that O_2_^•−^ was causing most of the oxidative damage of complex I. Altogether, these results challenge the notion that proline has a prominent function as an antioxidant and suggest that it does not provide protection against superoxide.

Contrary to the direct scavenging hypothesis, the possibility that proline may indirectly eliminate ROS, particularly by protecting antioxidant enzymes and increasing their activity, seems more likely and convincing. Indeed, a number of studies have reported that proline induces the activity of antioxidant enzymes such as catalase (CAT), peroxidase, polyphenol oxidase, and ascorbate peroxidase (APX) [13,49,87], suggesting that some of the observations of proline amelioration of oxidative damage may be related to an enhancement of the enzymatic antioxidant machinery. The stimulating effect of proline on the antioxidant defense could either be mediated by the kosmotropic or osmoprotective properties of proline or by direct or indirect induction of the expression of the genes of antioxidant enzymes.

### 3.4. Proline as Signaling Molecule

Under non-stressed conditions, homeostatic levels of proline are maintained by feedback regulation of P5CS activity, as well as reciprocal regulation of gene expression of *P5CS* and *ProDH*, indicating that plants sense and respond to intracellular proline concentration [37,88]. External proline application induced 85 out of 7000 analyzed *Arabidopsis* transcripts and a rehydration- and proline-responsive CIS-element (ACTCAT) was identified in the promoter of *ProDH* and other proline-responsive genes [89,90]. Members of the bZIP family of transcription factors have been identified as mediators of hypo-osmolarity-induced induction of ACTCAT-containing promoters [91,92]. Transcriptome analysis of chromium stressed *Arabidopsis* seedlings revealed that the beneficial effect of additional proline treatment was correlated with the differential expression of genes encoding proteins involved in cell wall biosynthesis and DNA repair [93,94]. Together with the data on the induction of the antioxidant defense system, these observations show that a significant part of the functions of proline could be mediated by specific signal transduction. However, the molecular mechanisms of proline sensing and signaling and the full range of target genes remain to be discovered.

### 3.5. Proline Metabolism

Despite the clear correlation between proline accumulation and its beneficial effects under stress, growing evidence suggests that proline metabolism, rather than proline itself, plays a crucial role in the impact of proline on stress responses and development [49,51,95,96]. As originally proposed by Hare and Cress [51], and described above, in most plant species the pool of free proline that accumulates in the cytoplasm during and after stress appears to be of insufficient size to account for the beneficial effects of proline on stress tolerance (see also Forlani et al. [33]). This observation implies that proline accumulation per se may not be the cause of proline-induced stress resistance to environmental stress. Second, an increasing number of papers report a lack of correlation between proline levels and stress tolerance or interpret the positive effects of proline against stress in terms of proline metabolism [13,49,53,58,96,97,98,99,100]. This controversial finding suggests that the simple accumulation of proline may not be sufficient to fully explain the adaptive response of proline under stress, prompting the search for other mechanisms.

In contrast, the unique characteristics of proline metabolism and its central role in intermediary metabolism may better explain its beneficial effects under stressful conditions. An important feature of proline metabolism is that its synthesis occurs in the cytosol, where it is either used for protein synthesis or transported to other cell compartments or tissues. According to some authors [8], proline synthesis could also occur in the chloroplast, especially under stress conditions, but this statement is not consistent with the absence of a chloroplast transit peptide in P5CS and is not confirmed by more recent work (see Trovato et al. [37] for more details). For degradation, proline is imported by unknown carriers or transporters into mitochondria. Both proline synthesis from glutamate and proline catabolism to glutamate share the same intermediate, P5C, which is in tautomeric equilibrium with GSA. As noted by Phang [101,102], proline and P5C are not interconverted by a single reversible enzyme but by two different enzymes with distinct mechanisms. A cycle between cytosolic P5CR and mitochondrial ProDH has been proposed to act as a redox shuttle in animal cells and promote ROS formation in mitochondria without net proline degradation [103,104]. Miller et al. [105] proposed the activity of such a cycle also in plants, but it is not yet clear whether P5C is exported in substantial amounts from mitochondria or whether it is primarily converted to glutamate by P5CDH. Several carriers were identified that mediate transport of glutamate across the mitochondrial membrane, thus cycling between proline and glutamate is definitely feasible if the enzymes of proline biosynthesis and catabolism are active at the same time [106,107]. The metabolic cycling between proline and P5C via ProDH and P5CR may provide a mechanism for transferring reducing equivalents from NAD(P)H into mitochondria, helping to maintain the balance of NAD(P)^+^ and NAD(P)H in the cytosol, especially when excess proline saturates the capacity of P5CDH to oxidize P5C to glutamate.

As for proline synthesis, the biosynthetic pathway from glutamate consumes two molecules of NADPH and one molecule of ATP per molecule of proline (Figure 1), with important implications in the reduction of stress-induced acidosis caused by NADPH accumulation. Even more important, the oxidation of NADPH to NADP^+^ stimulates the activity of the oxidative pentose phosphate pathway, which is essential for the synthesis of nucleotides and nucleic acids, the synthesis of aromatic acids and polyphenols, and the regeneration and sustenance of the ascorbate/glutathione pathway for ROS scavenging [51,108]. Additionally, if shuttled into chloroplasts, the NADP^+^ could be used as a terminal electron acceptor in the photosynthetic electron transport chain, reducing the risk of photoinhibition and of damaging the photosystem II (PSII) complex.

As for proline catabolism, the oxidation of proline to P5C and glutamate yields reducing equivalents in the form of FADH_2_ and NADH, which can be fed into the mitochondrial electron transport chain to produce ATP, thereby providing energy for the cell. This process is extremely efficient, as the oxidation of one molecule of proline to glutamate yields approximately 5 ATP equivalents, and complete oxidation of proline via glutamate and the tricarboxylic acid cycle yields approximately 30 ATP equivalents [109]. Proline catabolism is crucial for energy-consuming processes such as recovery from stress [110], root elongation [12,67], and the initial flight phase in some insects [111,112]. Due to the presence of glutamate dehydrogenase (GDH) in the mitochondrial matrix, glutamate may be converted to α-ketoglutarate (AKG) and fed into the mitochondrial TCA cycle.

Overall, the importance of anabolic and catabolic pathways in plant cell metabolism and the ability to control the balance between NAD^+^/NADH and NADP^+^/NADPH as a redox buffer through the proline-P5C cycle may well explain the beneficial effects of proline under and after stress.

## 4. Proline in Plant Development

**Figure 3:**
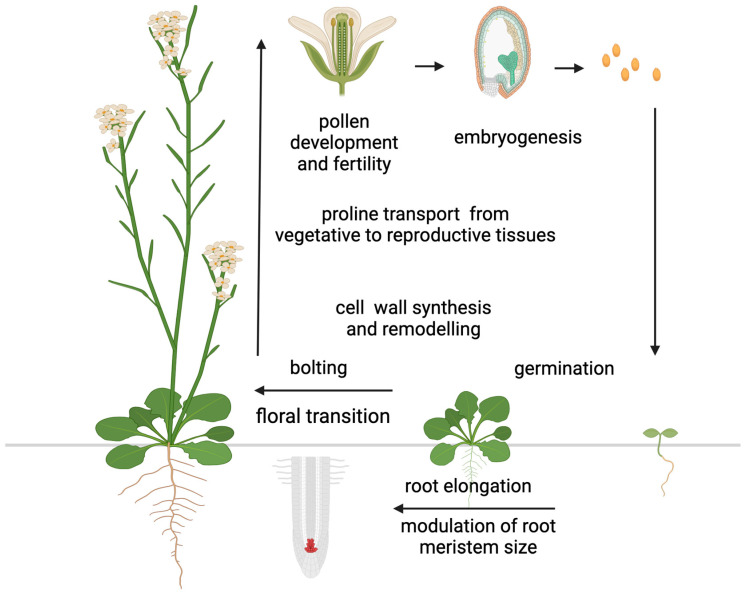
Proline functions in plant development. The diagram illustrates the main functions and developmental processes proline is involved in. Most of these functions are related to the reproductive stage, with the exception of germination and root elongation. Figure created with BioRender.com.

In addition to accumulating in response to biotic and abiotic stress, large amounts of proline have been detected in the reproductive organs of some plant species under non-stressed conditions. Specifically, high proline concentrations have been detected in pollen grains of petunia (*Petunia hybrida*), cowpea (*Vigna unguiculata*), and tomato (*Lycopersicum esculentum*) [113,114,115,116], ovules of broad bean (*Vicia faba*) [117], inflorescences and siliques of rapeseed (*Brassica napus*) [118], flower buds and young pods in barrel medic (*Medicago truncatula*) [119], and seeds in thale cress (*Arabidopsis thaliana*) [120], suggesting a role for proline in plant development (Figure 3).

The most convincing example of this evidence came from the excellent work of Chiang and Dandekar [121], who found that the percentage of proline relative to the total amino acid pool increased from 1–3% in the vegetative rosette of *Arabidopsis* prior to floral transition to about 26% in the reproductive tissue after floral transition. Schwacke et al. [116] reported a similar finding in *Lycopersicum esculentum*, where the proline content in flowers was 60 times higher than in any other organ analyzed. Although the striking difference in proline concentrations between vegetative and floral tissues found in tomato (*Lycopersicum esculentum*) is exceptional, a remarkable remobilization of proline from vegetative to floral tissues after floral transition is widespread among plant species, suggesting a special role for proline in floral transition and flower development. A complex developmental regulation underlies the redistribution of proline after floral transition involving long-distance transport [122], active transport between different cell compartments [116,120,123,124], direct synthesis within target tissues [121,125], selective catabolism [126,127], as well as the rate of protein synthesis and protein degradation [128]. However, despite the number of papers reporting accumulation of proline under normal conditions to much higher concentration than other proteinogenic amino acids, this phenomenon has long been overlooked. A major step in understanding the role of proline in plant development came from the genetic manipulation of the short proline pathway, particularly *P5CS1* and *P5CS2*, the paralog genes encoding the enzyme that controls the rate-limiting steps of proline synthesis in *Arabidopsis*. The study of plants transgenic for proline metabolism genes and *Arabidopsis* mutants defective in proline synthesis or degradation has helped to uncover a role for proline in germination [129,130,131], floral transition [2,3,4,5,47], embryo development [8,9], pollen fertility [6,7,125], and root development [12,13,132]. Moreover, in the cell wall matrix, proline and hydroxyproline are major constituents of proteins and glycoproteins, such as extensins, arabinogalactans, and proline-rich proteins [133,134,135]. These proteins are essential for cell wall synthesis and remodeling and play crucial roles in cell elongation, division, and differentiation [10,136], highlighting the importance of proline in plant development (Figure 3).

The first evidence supporting the role of proline in normal plant development was likely provided by the work of Kavi Kishor et al. [47], who described increased root biomass and flower development in transgenic tobacco overexpressing *P5CS*. Later, Nanjo et al. [2] generated transgenic *Arabidopsis* for a *P5CS* gene in antisense orientation, which showed morphologically altered leaves and stunted inflorescences. In addition, Samach et al. [3] identified *P5CS2* as an early target of *CONSTANS* (*CO*), a transcription factor that promotes flowering in Arabidopsis in response to day length. Collectively, these early findings suggested a role for proline in bolting and flower development. Consistent with these observations, Mattioli et al. [4] reported that CaMV35S-driven overexpression of *P5CS1* led to early flowering and bolting during the initial stages of plant development in *Arabidopsis*, although, in the later stages, reduced proline levels and a bushy appearance were observed, likely due to homology-driven gene silencing of both *P5CS1* and *P5CS2* [6]. Supporting these findings, a partial double mutant homozygous for *P5CS1* and heterozygous for *P5CS2* (hereafter referred to as *p5cs* sesquimutant) contained less proline than the wild type and exhibited late flowering, probably due to the upregulation of *FLOWERING LOCUS C* (*FLC*), a master repressor of flowering in *Arabidopsis* [5,9]. In the *p5cs* sesquimutant the effects of the two parental mutants on flowering time are additive, since the *p5cs* sesquimutant flowers later than the two single mutants. However, the effects of the mutations on Arabidopsis development are very different, indicating largely non-redundant functions of *P5CS1* and *P5CS2* [8,40] with *P5CS1* mediating stress-induced proline accumulation and *P5CS2* more involved in plant development, particularly embryo development and pollen fertility [6,7,8,9,125]. More recently, by physiologic and metabolic analyses, Park et al. [137] found that the Ca^++^-induced flowering delay in peach (*Prunus persica*) is mediated by proline metabolism.

Regarding embryo development, two independent studies [8,9] have shown that embryos homozygous for a *p5cs2* mutation exhibit developmental arrest and a number of aberrations at different stages of embryo development, failing to develop into viable plants and suggesting a specific role for *P5CS2* in embryogenesis. Confirming the specific effect of proline in embryo development, proline supplementation was shown to complement this lethal defect, although different authors report different levels of complementation. Mattioli et al. [9] reported only partial complementation, while Székely et al. [8] were able to rescue whole but abnormal and infertile plants. Funck et al. [6] reported that in vitro culturing of immature homozygous *p5cs2* embryos yielded fertile plants that produced viable seeds without further proline supplementation under short-day conditions. In addition to the critical role of *P5CS2*, *P5CR* is also required for embryo development, as homozygous *p5cr* mutants exhibit embryo lethality, with embryos aborting after only a few cell divisions [6]. Interestingly, while *P5CR* expression is essential for embryo development, it is not required for pollen or egg cell fertility, unlike *P5CS2* (see next paragraph), likely due to the extreme stability of the P5CR protein [6].

In addition, Dourmap et al. [138] have recently reported a novel role for P5CDH in reallocating carbon and nitrogen from the stem to the seed, which is critical for accumulating the reserves required for seed development. They found that mutants defective in P5CDH activity produce small seeds with low carbon and nitrogen content and exhibit low germination rates, particularly under high nitrate availability. It is currently unknown whether the small size and wrinkled appearance of *p5cdh* mutant seeds are caused by small embryo size or a lack of reserve material. As P5CDH is involved in the degradation of both proline and arginine, the defects in *p5cdh* mutants may derive from the block in either one or both of these pathways.

Regarding pollen fertility, Funck et al. [6] and Mattioli et al. [7] independently found a novel role for proline in pollen development by crossing *p5cs* sesquimutant plants with the wild type. When the *p5cs* sesquimutant was used as the female, approximately 50% of the offspring contained a *p5cs2* allele, as expected. However, less than 1% of the progeny received a *p5cs2* allele when the *p5cs* sesquimutant was used as the pollen donor. Morphological and functional analysis of the mutant pollen revealed a group of small, degenerated, and non-viable pollen grains mixed with normal-looking pollen grains, confirming that the *p5cs* sesquimutant is defective in pollen development and suggesting that proline biosynthesis plays a vital role in male gametophyte development.

Proline accumulation has also been positively correlated with seed germination, although the evidence remains limited, and a definitive demonstration of proline’s role in germination is still awaited. Several studies have reported improved germination rates under stress in transgenic tobacco plants overproducing proline by expressing *ornithine-δ-aminotransferase* [129] or *P5CS* [139]. Hare et al. [130] demonstrated that plants expressing an antisense version of *P5CS* exhibited low proline content and impaired germination, which could be partially rescued by exogenous proline supplementation. Furthermore, they observed a four-fold increase in proline levels in most *Arabidopsis* seeds prior to radicle emergence. This increase was correlated with an activation of rate-limiting enzymes in the OPPP, leading to the hypothesis that the OPPP plays a significant role in seed germination by providing the necessary reducing power and metabolic intermediates required for biosynthetic processes during early seedling growth [130,140]. Furthermore, as described for embryo development, the inability of *p5cdh* mutants to oxidize P5C to glutamate negatively affects germination, supporting a correlation between proline (or arginine) metabolism and germination [138].

In addition to reproductive development, recent reports [13,132] have revealed the importance of proline in root development. This novel function is not completely unexpected, as the elongation of hairy roots induced by transformation with *Agrobacterium rhizogenes* was initially attributed to the action of *rolD* contained on the T-DNA [141,142]. *rolD* was later identified as *ornithine cyclodeaminase* (*OCD*), a gene of bacterial origin coding for an enzyme that directly converts ornithine to proline [12]. In addition, a specific requirement for proline metabolism has been reported in the elongation of primary roots of maize and Arabidopsis under low water potential conditions [11,95]. Later, proline was found to modulate root meristem size in *Arabidopsis* by affecting cell division and controlling the ratio between cell division and cell differentiation [9,132].

Although most of the known effects of proline on plant development have been found in *Arabidopsis*, the conservation of its biochemical pathway and role in stress defense across plant species suggests that these effects are likely to be generalizable to most plants. However, further research and experimental confirmations are needed to fully validate this hypothesis across a broader range of species.

## 5. Proline–ROS Interactions

Intriguingly, the effects of proline on root meristem size were found parallel to and independent from auxin, cytokinin, and gibberellic acid (GA) pathways and not involving genes controlling cell differentiation at the transition zone, such as *ARR1*, *ARR12*, and *SHY2* [132]. Subsequent work has shown that proline metabolism affects the levels and distribution of superoxide anion (O_2_^•–^) and hydrogen peroxide (H_2_O_2_), which in turn modulate root meristem size and root elongation [13]. This finding was in agreement with Foreman et al. [143], who had previously shown that ROS produced by NADPH oxidase regulate root elongation through the activation of Ca^++^ channels, as well as with Dunand et al. [144] and Tsukagoshi et al. [145], who disclosed the relative role of O_2_^•–^ and H_2_O_2_ in root elongation. Bauduin et al. [13] found that exogenous proline concentrations at low µM levels induced root elongation and correlated with high levels of O_2_^•–^ and low levels of H_2_O_2_ in the root meristem, whereas high proline concentrations or proline-deficient mutants inhibited root elongation and correlated with H_2_O_2_ accumulation in the transition zone. Importantly, H_2_O_2_ scavenger treatment fully rescued the short root phenotype of the proline-deficient *p5cs* sesquimutant, as well as wild-type roots treated with high concentrations of proline. Furthermore, using mutants lacking ProDH activity, the same study revealed the critical role of ProDH in regulating root meristem size, probably as the main player in proline-induced O_2_^•–^ production in mitochondria. It is at present not fully clear how different concentrations of proline or different rates of proline degradation can have inverse effects on the ratio between O_2_^•–^ and H_2_O_2_ in different regions of the root meristem, especially since dismutation of O_2_^•–^ by SOD is presumably the predominant source of H_2_O_2_ [146]. In addition, crosstalk between ROS and auxin signaling in the regulation of root growth has been repeatedly observed [147,148], raising the unresolved question how the effects of proline can be mediated by ROS and at the same time be independent of auxin signaling. Interestingly, Bauduin et al. (2022) found that while proline itself may not directly control H_2_O_2_ levels, it appears to modulate the activity of catalase (CAT) and ascorbate peroxidase (APX), the two most potent H_2_O_2_ enzymatic scavengers in plant cells.

Although Bauduin et al. were unable to localize precisely the site of O_2_^•–^ in the root tip and limited their main conclusions to the effects of H_2_O_2_, their findings on the proline-mediated effects of O_2_^•–^ and H_2_O_2_ on root meristem size are largely consistent with the work of Dunand et al. [144] and Tsukagoshi et al. [145]. The latter authors identified the transcription factor UPBEAT1, which encodes a repressor of root peroxidases, and characterized mutant and overexpressor lines. Mutants deficient in *UPBEAT* showed low H_2_O_2_ and long roots, whereas *UPBEAT* overexpressors had high H_2_O_2_ levels and short roots. The authors could phenocopy the mutant phenotypes with exogenous H_2_O_2_ and complement the short root phenotype with scavengers of H_2_O_2_. Overall, they concluded that meristem proliferation in the root is determined by the balance between O_2_^•–^ and H_2_O_2_. Specifically, higher levels of O_2_^•–^ relative to H_2_O_2_ promote root elongation and meristem proliferation, while higher levels of H_2_O_2_ relative to O_2_^•–^ inhibit root elongation and reduce meristem size. Furthermore, they provided evidence that O_2_^•–^ is mainly located in the apoplast of the meristematic and elongation zones, whereas H_2_O_2_ is found in the transition and differentiation zones, and that the ratio between O_2_^•–^ and H_2_O_2_ determines root meristem growth in a hormone-independent manner.

To confirm the interactions between proline and H_2_O_2_ at the genetic level, Bauduin et al. [13] crossed the *p5cs* sesquimutant with *upb1* [145]—a mutant allele of the transcription factor UPBEAT—whose inactivation leads to increased peroxidase activity and low levels of H_2_O_2_, resulting in roots longer than wild type, and analyzed the meristem size of the resulting *p5cs upb1* quasi-triple mutant. The root meristem size of *p5cs upb1* was intermediate between *p5cs* and *upb1* root meristems, suggesting that the effects of H_2_O_2_ on root length are dose-dependent and that the balance of H_2_O_2_ levels is crucial for determining root meristem size. This finding corroborates the link between proline and ROS and supports the idea that root meristem size and root elongation are regulated by a delicate balance between O_2_^•–^ and H_2_O_2_ levels, and that both proline synthesis and peroxidase activity play significant, although independent, roles in modulating this balance.

Since ROS are highly reactive and potentially toxic molecules that are constantly produced by the cell during normal metabolism and in response to environmental stresses, they are tightly controlled by a dynamic and complex array of enzymatic and non-enzymatic antioxidants, presumably including proline. Indeed, some non-enzymatic antioxidants such as glutathione [149], catechol [150], melatonin [151], and gallic acid [152] have been shown to modulate root meristem growth by controlling ROS levels. The correlation between H_2_O_2_ accumulation and the short root phenotype of the *p5cs* sesquimutant, as well as the inhibition of root growth induced by high concentrations of H_2_O_2_ (or proline), is easily understood given the known cellular toxicity of hydrogen peroxide [153,154]. It is well known that high concentrations of hydrogen peroxide, as well as other ROS and reactive nitrogen species (RNS), can be toxic and even lethal to plant cells. They can cause lipid peroxidation leading to membrane damage and loss of cell integrity, protein oxidation with serious consequences for enzyme activity and metabolic processes, and irreversible DNA damage and modification affecting gene expression and signaling pathways [154].

It is less intuitive to understand the stimulatory effects on root growth induced by low concentrations of proline (or H_2_O_2_). However, although earlier research focused mainly on ROS toxicity, current research has well established that ROS have a dual role in plants: toxic at high concentrations and beneficial at low concentrations, where they act as important signaling molecules that regulate normal plant development and responses to stress [17,155,156,157]. These latter processes involve interactions between NADPH oxidases, calcium (Ca_2_^+^) channels, and oxidative stress-induced Ca_2_^+^ fluxes. Intriguingly, proline-mediated Ca_2_^+^-dependent signaling was recently reported in the regulation of carbon/nitrogen metabolism of rice (*Oryza sativa*) subjected to Cr(VI) stress [158]. Similarly, by physiologic and metabolic analyses, Park et al. [137] showed that the Ca_2_^+^-induced late flowering in peach (*Prunus persica*) is dependent on proline metabolism. Overall, these results suggest a potential crosstalk between proline and ROS signaling pathways.

Proline–ROS interactions have also been described in response to biotic [159] and abiotic stress [160,161]. However, in contrast to unstressed conditions, where it seems to act mainly as a pro-oxidant, stress-induced proline accumulation or suppression of proline degradation seems to promote antioxidant activities. In a study investigating the basis of the hypersensitive response elicited by *Arabidopsis* in response to infection by an avirulent race of *Pseudomonas syringae*, Fabro et al. [159] found a positive correlation between ROS and proline. The authors observed a local increase in reporter activity in transgenic *P5CS2:GUS* or *P5CS2:LUC* plants infiltrated with a superoxide generator, suggesting that ROS can trigger the activation of *P5CS2* and lead to proline accumulation during biotic stress. Additionally, under abiotic stress, several papers reported correlative evidence of ROS-induced proline accumulation, as reported by Uchida et al. [160], who found an upregulation of P5CS in rice seedling leaves in response to H_2_O_2_ treatment. Similarly, Yang et al. [162] described a significant accumulation of proline in coleoptiles and radicles of maize seedlings treated with H_2_O_2_, caused by the simultaneous increase in P5CS activity and decrease in ProDH activity. In later studies, Ben Rejeb et al. [161] investigated the relationship between ROS and proline and convincingly demonstrated that H_2_O_2_ generated from NADPH oxidase-produced O_2_^•–^ leads to proline accumulation in *Arabidopsis thaliana* plants treated with 200 mM NaCl or 400 mM mannitol. Similarly, Liu et al. [163] reported that NADPH oxidase-mediated H_2_O_2_ reduced salt stress in wheat seedlings by enhancing proline accumulation.

**Figure 4:**
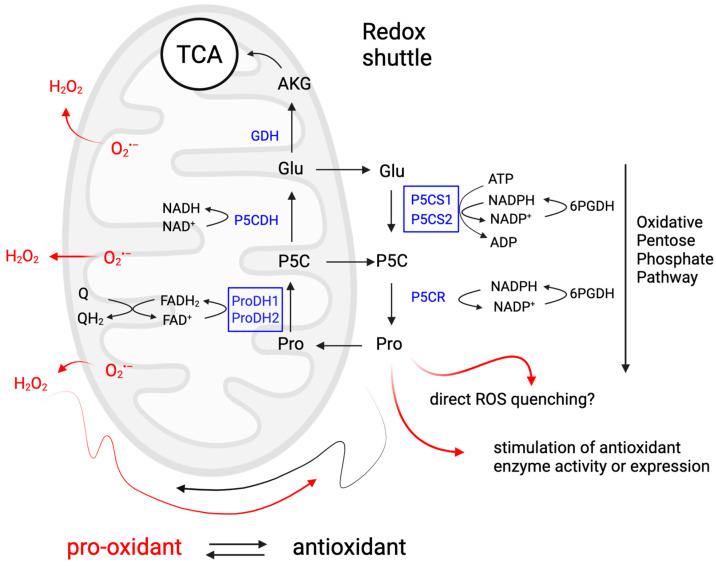
Proline metabolism as a redox hub in plant response to stress and developmental stimuli. This diagram illustrates how proline metabolism can act as a redox shuttle between cytosol and mitochondria and modulate ROS homeostasis. Proline catabolism generates ROS in mitochondria and acts as a pro-oxidant, while proline synthesis lowers the cytosolic NADPH/NADP+ ratio, which stimulates the oxidative pentose phosphate pathway. Proline accumulation acts as an antioxidant by scavenging ROS, protecting membranes and organelles from ROS through its kosmotropic properties and by activating antioxidant enzymes. Through these mechanisms, proline metabolism can alter both the levels and species composition of ROS, which allows plants to integrate and respond to different stimuli and has the potential to explain most of the functions attributed to proline. Figure created in BioRender.com.

Overall, these studies highlight the importance of ROS in plant development and stress responses, emphasizing the need to control their levels. Thanks to its ability to (re)generate ROS and ATP in the mitochondrion via the proline–P5C or proline–glutamate cycle between the mitochondrion and cytosol, and the feedback loop between antioxidant activity during proline synthesis and pro-oxidant activity during catabolism, proline can control root development by regulating ROS levels and distribution in the root meristem (Figure 4).

## 6. Can Interactions with ROS Explain Other Functions of Proline in Stress and Development?

It is well known that all stresses, both biotic and abiotic, generate different types of ROS in various locations [38,164,165]. In parallel, proline accumulates in most plants in response to stress, likely participating in and stimulating an antioxidant response. Accordingly, the interactions between proline metabolism and ROS, along with the consequent control of cellular redox balance and activation of signaling pathways to trigger antioxidant responses, may be considered a general or widespread mechanism of stress response. Other possible mechanisms, on the contrary, seem to be linked to specific situations. The accumulation of proline as a possible osmoregulation factor, for example, could be justified in response to salt or drought stress but seems doubtful in response to stresses without an osmotic component, such as nutritional stress, heavy metal stress, mechanical stress, and biotic stress from pathogen attack.

It is therefore likely that most, if not all, early responses to biotic or abiotic stress involve an interaction between proline and reactive oxygen species (ROS). However, does this also apply to all developmental processes modulated by proline, or only to the fine tuning of root meristem size?

### 6.1. Germination

As to germination, it is well documented that ROS play a pivotal role in regulating seed dormancy, germination, and viability [166,167]. In dry seeds, ROS are generated through non-enzymatic reactions, such as lipid auto-oxidation [168]. However, as soon as seed imbibition starts, enzymatic reactions begin to prevail [169,170]. Low levels of ROS act as signaling molecules that promote the release of physiological dormancy and trigger seed germination [171,172], by promoting endosperm weakening [173], stimulating gibberellic acid (GA) synthesis, and reducing abscisic acid (ABA) levels [174,175]. In contrast, high levels of ROS typically lead to seed deterioration by affecting lipid peroxidation, membrane permeability, protein integrity, and mitochondrial function [166]. Another important role of H_2_O_2_ in seed germination is the activation of α-amylase and the induction of programmed cell death (PCD) in the aleurone layer, in cooperation with the GA signaling repressor DELLA proteins [174,176]. Additionally, lower viability in long-term stored common bean (*Phaseolus vulgaris*) seeds has been shown to correlate with higher contents of O_2_^•–^, H_2_O_2_, and •OH [177]. In agreement with this finding, Trovato et al. [178] observed low viability associated with high contents of O_2_^•–^ and H_2_O_2_ in four-year stored seeds of *p5cs* sesquimutants compared to wild-type seeds of the same age. In addition, the low germination rate of *p5cdh* mutants, as a consequence of impaired carbon and nitrogen remobilization during seed filling [138], could potentially be triggered by ROS signaling. This is suggested by the increase in ROS production exhibited by *p5cdh* plants infected with avirulent strains of *Pseudomonas syringae* [159]. Without P5CDH to convert P5C into glutamate, P5C may damage mitochondria and stimulate ROS formation [179]. Alternatively, P5C may be exported from mitochondria and be converted back to proline by P5C reductase (P5CR), return to the mitochondrion, and be oxidized again to P5C [20]. In this proposed Pro/P5C cycle, O_2_^•–^ could be continuously produced as a by-product without net proline consumption (Figure 4).

### 6.2. Pollen Development and Pollination

Although at present there are no reports showing a direct link between proline and ROS in pollen development and fertility, indirect evidence strongly suggests that this might indeed be the case. In fact, ROS are essential in various stages of pollen development and fertilization, including tapetum degeneration, pollen tube growth, and pollen–stigma interactions [180,181,182], but because of their potential toxicity, they must be tightly regulated by enzymatic and non-enzymatic antioxidants. Since proline has been found in large amounts in pollens of several plant species at different stages of development [183], it is a strong candidate to play a major role as a ROS antioxidant in the pollen grain, as well as an important energy source.

Consistently, insufficient amounts of proline in pollen caused by mutations in proline synthesis genes lead to pollen degeneration and male sterility, indicating that proline accumulation in pollen grains is essential for their viability and fertility [6,7,125].

In addition, proline accumulation in response to environmental stress may play a critical role in protecting pollen grains by modulating ROS levels that are inevitably associated with environmental stress. It is well known that flowering plants are highly sensitive to abiotic stresses that severely impair sexual reproduction and reduce crop yield. This sensitivity is critically dependent on pollen viability, which is dramatically reduced by environmental stresses such as drought, salinity, and extreme temperatures [184]. Most of the yield reduction caused by these stresses is due to ROS accumulation, which leads to impaired anther and pollen development, incomplete anther dehiscence, reduced grain set, and precocious grain abortion [185,186,187,188]. Since both proline and ROS are crucial for pollen development and stress responses, it is plausible that proline may help modulate ROS levels to ensure proper pollen development and function under abiotic stress conditions. Accordingly, in wheat grown in the presence of 120 mM NaCl, pretreatment with proline increased grain yield and grain weight [189]. In maize treated with different concentrations of NaCl, proline solutions sprayed on maize leaves improved growth and grain yield at 25 mM and 50 mM NaCl [190]. Moreover, Mattioli et al. [56] reported that treatment with 150 mM NaCl administered after floral transition led to a significant reduction in the number of seeds per silique of wild-type Arabidopsis plants. However, when *p5cs* sesquimutants were subjected to the same treatment, the detrimental effect was more pronounced, with a significantly stronger reduction in the number of seeds per silique observed. Importantly, when a *p5cs* sesquimutant expressing an additional copy of the proline biosynthesis gene *P5CS2* under the control of the pollen-specific promoter of the *At5g17340* gene [191] was treated with 150 mM NaCl, the number of seeds per silique was significantly higher compared to the parental *p5cs* sesquimutant and close to wild type, supporting the hypothesis that proline may reduce salinity damage on seed production.

### 6.3. Female Gametophyte and Embryo Development

Despite the striking effect of proline deficiency on embryo development, leading to a number of morphological aberrations and ultimately embryo abortion [8,9], evidence for ROS involvement in plant embryogenesis is relatively scarce. ROS play a crucial role in the development and function of the female gametophyte in plants. They are involved in processes such as embryo sac patterning and polarity maintenance. ROS are also essential for pollen-pistil interactions, including pollen tube growth and fertilization [192,193]. Accordingly, the known role of proline as an antioxidant suggests that it could help modulate ROS levels and thereby protect the female gametophyte from oxidative damage during stress. In animal systems, however, proline has been shown to improve the development of preimplantation embryos by protecting them from oxidative stress. Studies in mouse embryos indicate that proline and its analogues can reduce mitochondrial activity and ROS levels, leading to improved embryo development and increased blastocyst formation [194], suggesting that the antioxidant properties of proline may play a role in maintaining redox balance during early embryo development. Overall, these findings support the hypothesis that proline–ROS interactions may be important for female gametophyte and embryo development, particularly under abiotic stress conditions, although further research is needed to establish direct links and fully understand the mechanisms involved.

### 6.4. Flowering Time

A number of studies report correlations between ROS and flowering time in the photoperiodic [195,196,197,198], GA-dependent [199,200], and stress-induced [201,202,203] pathways. Given the ability of proline to control the redox state and distribution of ROS in the plant cell, it is reasonable to speculate that proline may also influence flowering time through its interaction with ROS. Nevertheless, there are not many indications in the literature to support this view. One possibility is that proline-mediated variation in ROS accumulation may affect flowering time by controlling the expression of *FLOWERING LOCUS C (FLC)*, a master repressor of flowering time in *Arabidopsis* [204], whose expression is influenced by cellular redox status and ROS levels [198]. Indeed, it was recently reported that the *p5cs* sesquimutant, which is deficient in proline synthesis, shows an upregulation of *FLC* expression compared to wild-type plants [5]. Vernalization-induced down-regulation of *FLC* abolishes the flowering delay in these mutants, suggesting that *FLC* acts downstream of proline biosynthesis genes and is required for proline-modulated flowering. Since all proline synthesis mutants (*p5cs1*, *p5cs2*, and *p5cs1p5cs2/P5CS2*) accumulate H_2_O_2_ [8,9,13], it is reasonable to speculate that the altered redox balance due to proline deficiency leads to increased ROS levels, which in turn upregulate *FLC* expression and delay flowering.

Moreover, a floral pathway convincingly modulated by ROS, through interactions with the circadian clock and the FLAVIN-BINDING KELCH-REPEAT F-BOX1 (FKF1) transcription factor, is the photoperiodic pathway by which *Arabidopsis* regulates flowering time in response to day length. The CONSTANS (CO) protein acts as a master regulator of this pathway [205], integrating light signals and the circadian clock to regulate the expression of the *FLOWERING LOCUS T* (*FT*) gene [3,206]. Since proline has been found to oscillate with light and dark cycles due to the circadian regulation of the enzymes P5CS and ProDH [207], and P5CS2 has been shown to be one of the early targets of CO [3], the involvement of proline in the photosynthetic pathway, through interactions with ROS and the circadian cycle, is conceivable.

## 7. Conclusions and Future Perspectives

As described above, most of the mechanisms hypothesized to account for the beneficial effects of proline, both in response to biotic or abiotic stresses and in plant development, are related to ROS or redox balance, suggesting a universal role for proline metabolism in controlling plant responses through regulation of ROS homeostasis (Figure 4). Can we therefore consider the redox buffering properties of proline metabolism and its role in maintaining ROS homeostasis as a unifying mechanism underlying all known functions of proline in plant development and stress defense?

Perhaps not, at least not yet. Despite the remarkable physicochemical properties of this amino acid, which could potentially explain all the functions attributed to proline in terms of ROS modulation and redox buffering, we currently lack sufficient evidence to support such a general claim. However, this review suggests that proline metabolism, together with a battery of enzymatic and non-enzymatic antioxidants, participates in the complex regulatory system that serves to regulate and control ROS signaling. Because of its antioxidant and pro-oxidant properties, proline metabolism is involved in modulating redox balance and ROS homeostasis, which could potentially explain many of the multiple roles attributed to proline and deserves further investigation. There are still many gaps in our knowledge regarding the relationships between proline and ROS that warrant further investigation. First, we should study the role of proline metabolism, if any, in all the physiologic processes proline is involved in, and improve our knowledge of those in which the interactions between ROS and proline are already known. For example, we still lack understanding of the downstream genes or the signaling pathways involved that respond directly to proline or to proline metabolism-derived ROS stimuli in the root meristem to adjust meristem size to environmental and developmental variations. Even more complex, and deserving of further investigation, is the synergy between proline metabolism and ROS homeostasis during sexual reproduction, from the development of male and female gametophytes to the formation of a viable embryo, of which we currently have only a fragmented and incomplete picture. Another important question that we may want to address in the future is how the multiple players that contribute to the control and regulation of redox balance and ROS signaling—the enzymatic and non-enzymatic antioxidants—communicate with each other, and likely also with plant hormones, to produce integrated responses. It is still unclear how proline can control synthesis and catabolism to tailor ROS production and removal to specific tasks, nor do we know how much the proline-P5C or proline-glutamate cycles contribute to this mechanism and the details of the signal perception/transduction pathways upstream of proline metabolism.

Furthermore, in a global context characterized by increasing temperatures and droughts, especially in some geographical areas, there is a growing interest in finding biotechnological solutions to accelerate the adaptation of crops to new environmental conditions [208,209,210]. A better understanding of the interactions between proline and ROS and between enzymatic and non-enzymatic antioxidants promises to have important applicative implications.
